# Evaluation of Individual and Combined Markers of Urine Dipstick Parameters and Total Lymphocyte Count as a Substitute for CD4 Count in Low-Resource Communities in Ghana

**DOI:** 10.1155/2018/7485942

**Published:** 2018-02-11

**Authors:** Enoch Odame Anto, Christian Obirikorang, Emmanuel Acheampong, Bright Amankwaa, Bright Oppong Afranie, Sampson Donkor, Isaac Hope, Juliana Jommo, Esther Osaah

**Affiliations:** ^1^Department of Molecular Medicine, School of Medical Sciences, Kwame Nkrumah University of Science and Technology, Kumasi, Ghana; ^2^School of Medical and Health Sciences, Edith Cowan University, Perth, WA, Australia; ^3^Laboratory Unit, Saint Francis Xavier Hospital, Assin Fosu, Ghana; ^4^Department of Medical Laboratory Science, Royal Ann College of Health, Kumasi, Ghana

## Abstract

We evaluated the individual and combined levels of urine dipstick and total lymphocyte count (TLC) as surrogate markers for CD4 count in a low-resource community in Ghana. This cross-sectional study recruited 200 HIV-infected patients from the Saint Francis Xavier Hospital, Assin Fosu, Ghana. Complete blood count, CD4 count, and urine dipstick analysis were measured for participants. The threshold values were determined as <350 cells/*μ*l for CD4, <1200 cells/*μ*l for TLC, and ≥+ on urine dipstick analysis. The mean age of participants was 43.09 years. Proteinuria ≥ + [aOR = 4.30 (3.0–18.5)], leukocyturia ≥ + [aOR = 2.91 (1.33–12.5)], hematuria ≥ + [aOR = 2.30 (1.08–9.64)], and TLC < 1200 cells/*μ*l [aOR = 3.26 (3.94–15.29)] were significantly associated with increased risk of CD4 count < 350 cells/*μ*l. Using the individual markers, the best substitute marker for predicting CD4 count < 350 cells/*μ*l was proteinuria at a cutoff point ≥ 2++, AUC of 0.973, sensitivity of 97.6%, specificity of 100.0%, PPV of 100.0%, and NPV of 89.1%. A combination of ≤ 1200 TLC + ≥ 2++ (leukocyturia + proteinuria + hematuria) yielded an AUC of 0.980, sensitivity (72.8%), specificity (100.0%), PPV (100.0%), and NPV (97.9%). Proteinuria could serve as a noninvasive screening tool, but the combination of proteinuria, leukocyturia, hematuria, and TLC serves as a better substitute marker for CD4 count in monitoring the disease progression among HIV patients in low-resource communities.

## 1. Background

Human immunodeficiency virus (HIV) remains a public health burden in both developed and underdeveloped countries [1]. Globally, 37 million people are living with HIV, out of which about 26 million of them reside in sub-Saharan Africa [2]. In Ghana, the prevalence of HIV among adult aged 15–49 years old has declined from about 2.4% in 2013 to about 1.6% in 2015 [3]. The rapid use of highly active antiretroviral therapy (HAART) in the last eight years especially in developing countries has yielded tremendous achievements and improvement of CD4 counts of immune compromised individuals. The previous guidelines recommended to start HAART for HIV confirmed individuals with CD4 count < 200 cells/*μ*l. The current improvements in diagnosis and treatments have led to the development of a new treatment guideline which recommends HAART for all confirmed HIV individual with CD4 count 350 cells/*μ*l to 500 cells/*μ*l depending on the clinical conditions and comorbidities [4, 5]. Despite this achievement to treatment, most developing countries have inadequate facilities, inadequate CD4 FASC count machines, inadequate human resources, and insufficient supply of CD4 count reagents [6]. In addition to the aforementioned challenges, Ghana still faces a huge financial crisis needed to support HIV treatment and repair broken-down FASC count machine [7]. For the reason that Ghana has not yet implemented the latest WHO guidelines, means implementing it is likely to increase enrolment of participants for HAART and also worsen the challenges faced by the low-resource communities [8].

The WHO recommendation that ART should commence for all HIV-confirmed individuals in clinical stages II or III with total lymphocyte count (TLC) < 1200 cell/*μ*l was also in recognition to the challenges faced by low-resourced communities [9]. Following this initiation, some studies have found a significant correlation between CD4 count and TLC [10–12]. The use of TLC yielded a high sensitivity and specificity in predicting CD4 count < 200 cell/*μ*l among HIV-confirmed patients living in Ghana [13] and Ethiopia [14]. However, other studies by Daka et al. [12] and Akinola et al. [15] found TLC as a poor diagnostic marker and hence could not recommend it as a substitute for CD4 count. To overcome this limitation, some studies explored the diagnostic performance of TLC in combination with other less expensive parameters such as hemoglobin, hematocrit, BMI, and erythrocyte sedimentation rate (ESR) [11, 16]. These studies observed a better sensitivity and specificity of the combined markers for predicting CD4 count < 200 cell/*μ*l compared to using the individual markers alone. Although, most of the markers used in the latter studies were less expensive, they required adequate expertise and extensive electricity power supply. Hence, there is a need to develop a much lesser expensive marker as a substitute for CD4 count in these settings [17].

Noninvasive markers of dipstick urine abnormality findings such as proteinuria, leukocyturia, and hematuria were found to be significantly associated with a declined CD4 count [18]. To date, very few studies have attempted to confirm this finding. Whether the diagnostic performance of the dipstick urinalysis findings will be a useful substitute marker for CD4 count in a low-resource setting like Ghana is yet to be explored. Very little or no research has been conducted in Ghana among HIV-infected HAART naive individual after the implementation of the latest WHO cutoff point to start HAART. It is against this background that this study evaluated the individual and combined markers of urine dipstick findings and TLC as substitute markers for CD4 count < 350 cell/*μ*l in Ghana. Dipstick urinalysis is less expensive and will develop innovative approach to diagnosis and treatments, which will further facilitate implementation of the latest WHO treatment guideline.

## 2. Materials and Methods

### 2.1. Study Design, Period, and Setting

This was a hospital-based cross-sectional study carried out at the HIV Clinic of Saint Francis Xavier Hospital, Assin Fosu, in the Central Region of Ghana from August 2016 to May 2017. Saint Francis Xavier Hospital, Assin Fosu, is the largest hospital in the central region of Ghana with a 118 bed capacity and is a major referral centre that provides health services to patients from a catchment area population of 207,000 in Ghana [19].

### 2.2. Study Participants

Two hundred (200) newly diagnosed HIV-infected patients between the ages of 15–64 years at the HIV Clinic at Saint Francis Xavier Hospital were randomly selected for the study. Patients under antiretroviral therapy, pregnant women, and those with other medical conditions such as sickle cell disease, viral hepatitis B or C, diabetes mellitus, systemic lupus erythematosus, tuberculosis and acute viral infections, joint inflammatory diseases, sign of urinary tract infections were excluded from the study. Other signs of active infections and conditions that may interfere with urine dipstick analysis or kidney function test were also excluded. A questionnaire was used to collect sociodemographic characteristics of the study participants. Other vital information pertaining to the clinical history of all participants was obtained from the hospital's data review records for each patient.

### 2.3. Ethical Consideration

This study was approved by the Committee on Human Research Publications and Ethics (CHRPE) at the School of Medical Sciences, Kwame Nkrumah University of Science and Technology and the Ethical Committee of the at Saint Francis Xavier Hospital. Participation was voluntary and written informed consent was obtained from each participant. All information obtained from participants were kept under strict confidentiality.

### 2.4. Blood Collection, Total Lymphocyte, and CD4 Counts

A volume of 5 mL of venous blood was taken from the subjects via phlebotomy and transferred into vacutainer tubes with ethylenediaminetetraacetic acid (EDTA). Samples from the subjects were analysed within 2 to 4 hours of collection. The EDTA blood sample was used for the measurement of CD4 cell count and complete blood count (CBC). FBC was analysed using a five-differential automated blood analyzer (HORIBA Yumizen H500, Horiba Medical, Japan) and CD4 T lymphocyte count was determined using the Becton Dickinson (BD) FASCount system (Becton, Dickinson and Company, CA, USA). The BD FASCount system uses flow cytometry for the quantification of the CD4 T lymphocytes.

Total lymphocyte count was determined and expressed in cells/*μ*l as follows:

(1) $$\begin{array}{c} White\;blood\;cell\;\left(WBC\right)\times 1000\times percentage\;lymphocyte\;\left(expressed\;as\;decimals\right) \end{array}$$

### 2.5. Urine Collection and Biochemical Analysis

About 10–20 millilitres of early morning midstream urine were collected from each participant into a clean leak-proof container for analysis. Dipstick urinalysis strips (Accu-Tell Ref ABT-UM-A33) were used to detect the following parameters in urine provided by the participants: protein, glucose, bilirubin, urobilinogen, ketones, blood, and leukocytes.

### 2.6. Statistical Analysis

The data collected was entered into Microsoft Excel and analyzed with SPSS version 23. The basic characteristics were presented as means and standard deviations (SD) for continuous data and as frequencies for categorical data. Comparisons between male and female were made using independent Student's *t*-tests for continuous data and chi-square tests for categorical data. Receiver operating characteristic (ROC) curves were used to demonstrate the individual and combined diagnostic performance of selected urine dipstick biochemistry and TLC as alternative markers for CD4 count. Sensitivity, specificity, and positive and negative predictive values were reported. Logistic regression analysis adjusting for signs of active infections and conditions that may interfere with urine dipstick analysis was performed to predict urine dipstick biochemistry and TLC associated with CD4 counts. The level of significance was set at *p* < 0.05 for all statistical comparisons.

## 3. Results

The mean age of participants was 43.09 years, and the most frequently represented age group was 35–44 years (34.0%). There was no significant difference between the mean ages of males and females. There were more female participants (71.5%) than males (28.5%) and a higher proportion of the participants were divorced (56.0%) (Table 1).

As shown in Table 2, the general prevalence of leukocyturia, proteinuria, and hematuria was 50.0%, 54.5%, and 51.5%, respectively. There was no significant difference in proportion between males and females in terms of the presence and absence of leukocyturia (*p* = 0.3105), proteinuria (*p* = 0.8871), and hematuria (*p* = 0.2121). Additionally, no significant difference was observed between males and females in terms of the mean concentration of CD4 (*p* = 0.1109), TLC (*p* = 0.4017), Hb (*p* = 0.1992), and WBC (*p* = 0.3629).

There was a significant association of CD4 count with leukocyturia (*p* < 0.0001), proteinuria (*p* < 0.0001), hematuria (*p* < 0.0001), and TLC (*p* < 0.0001). Individuals with proteinuria (≥+), leukocyturia (≥+), hematuria (≥+), and TLC (<1200 cells/*μ*l) were significantly associated with 4.30 times, 2.91 times, 2.30 times, and 3.26 times increased risk of CD4 count < 350 cells/*μ*l, respectively, (Table 3).

Using various threshold points, the best cutoff point for leukocyturia was ≥2++ with sensitivity of 92.1%, specificity of 98.9%, positive predicted values (PPV) of 100.0%, and negative predicted value (NPV) of 83.3% for predicting CD4 count < 350 cells/*μ*l. The best threshold point for proteinuria was ≥2++ with sensitivity of 97.6%, specificity of 100.0%, PPV of 100.0%, and NPV of 89.1% for predicting CD4 count <350 cells/*μ*l. The best threshold point for hematuria was ≥2++ with sensitivity of 92.7%, specificity of 98.6%, PPV of 100.0%, and NPV of 83.7% for predicting CD4 count < 350 cells/*μ*l. TLC of <1200 was shown to have a sensitivity of 85.1%, specificity of 100.0%, PPV of 100.0%, and NPV of 88.9% for predicting CD4 count < 350 cells/*μ*l. The best area under the curve (AUC) was identified for proteinuria ≥ 2++ (AUC = 0.973) followed by TLC (AUC = 0.940), hematuria (AUC = 0.938), and leukocyturia (AUC = 0.901). The closeness of the AUC for proteinuria to “1.0” makes it a better diagnostic tool for predicting CD4 < 350 cell/*μ*l, hence making it a better alternative marker for CD4 count (Table 4).

As shown in Table 5, using the best threshold points <1200 cell/*μ*l for TLC, ≥2++ for proteinuria, ≥2++ for leukocyturia, and ≥2++ for hematuria, a sensitivity of 72.8%, specificity of 100.0%, PPV of 100.0%, and NPV of 97.9% was found to predict CD4 count <350 cells/*μ*l among HIV-infected participants.

**Figure 1:**
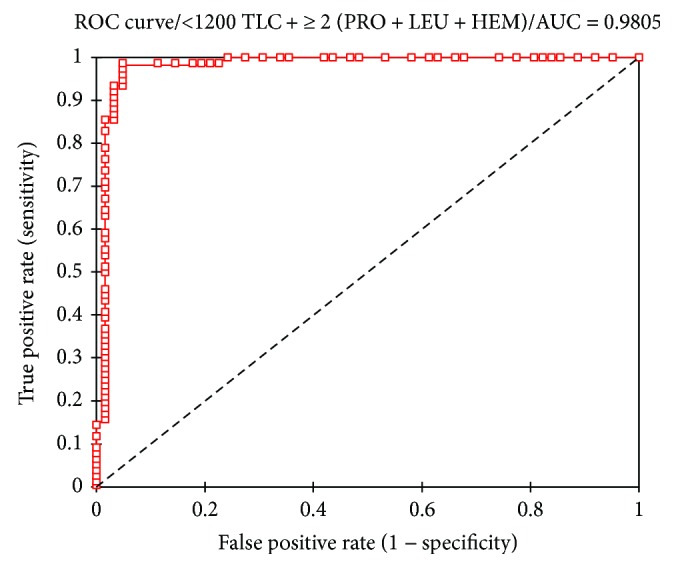
Receiver operating characteristic (ROC) curve for combined TLC, proteinuria, leukocyturia, and hematuria marker for predicting <350cells/*μ*l.

A shown in Figure 1, a higher AUC of 0.980 was observed for the combination of the significant threshold point for TLC, proteinuria, leukocyturia, and hematuria making the combined markers a perfect alternative marker for CD4 count.

## 4. Discussion

This study evaluated the individual and combined diagnostic performances of some selected measures of dipstick urinalysis and total lymphocyte count as alternative markers for CD4 count in low-resource communities in Ghana.

Based on our result, there was a significantly high proportion of hematuria, proteinuria, and leukocyturia among HIV-infected participants with a declined CD4 count. Significantly high counts of proteinuria, leukocyturia, and hematuria were independent risk factors for CD4 count < 350 cell/*μ*l. A cross-sectional study conducted by FolefackKaze et al. [20] among HIV-infected patients in Cameroon found significant associations of proteinuria, hematuria, and leukocyturia with a declined CD4 count, which is consistent with the findings in our study. The prevalence of proteinuria observed in this present study was significantly higher compared to the other urine abnormalities. This is consistent with previous reports that proteinuria is the leading urinary abnormality associated with declined CD4 count [20]. Therefore, renal insufficiency could be the underlining cause of proteinuria arising from an advanced stage HIV infection and a declined CD4 count [20–22]. Conversely, some studies found no significant association between renal failure and a decline in CD4 count under routine clinical management [23]. There is no clear mechanism linking a declined CD4 count to the presence of hematuria in HIV infection; however, this could be attributed to unforeseen cofounders [24, 25].

When TLC and the individual markers of dipstick urinalysis were explored for predicting CD4 count < 350 cells/*μ*l, proteinuria (at a cutoff point ≥ 2++) was observed as the best substitute for CD4 count with sensitivity of 97.6%, specificity of 100.0%, PPV of 100.0%, and NPV of 89.1%. Moreover, proteinuria when compared to TLC in this study was found to be the second-best substitute marker with a sensitivity of 85.1%, specificity of 100.0%, PPV of 100.0%, and NPV of 88.9% at a threshold value of <1200 cell/*μ*l. Our findings, which indicated that proteinuria, could serve as a better substitute marker for CD4 count is novel and first of its kind to be reported in a Ghanaian setting. In a cross-sectional study by Atta et al. [26], proteinuria yielded a sensitivity, specificity, and positive and negative predictive values of 73%, 61%, 53%, and 79%, respectively, for HIV-associated nephropathy. An increased proteinuria depicts decrease in glomerular filtration threshold or renal failure. This finding is also supported by the significant association between increased proteinuria and a decreased CD4 count observed in our current study. HIV-infected individuals with proteinuria ≥ 2++ were 4.30 times more likely to report with CD4 count below 350cells/*μ*l. Early diagnosis of proteinuria among HIV-infected patients will aid early detection of renal impairment, and this will form the basis to start HAART. Initiation of HAART has been shown to improve HIV-associated nephropathy [27].

In some studies, conducted by Obirikorang et al. [13] and Wondimeneh et al. [14], TLC was found to be the best substitute marker for predicting disease progression in HIV treatment naïve individuals with CD4 count <200 cell/*μ*l. In this study, TLC was found to be the second-best substitute marker for predicting CD4 < 350 cell/*μ*l. The disparity in findings could be attributed to the different CD4 count cutoff used. The use of CD4 count <350 cell/*μ*l is likely to enroll a majority of participants leading to an increasing statistical power and sample size compared to using CD4 count <200 cell/*μ*l. However, a cross-sectional study by Kamya et al. [28] found conflicting reports. Kamya and colleagues observed a 100% PPV but 32.0% NPV for TLC, which implied that TLC could limit the detection of HIV patients who are symptomatic with >1200 cell/*μ*l. Meanwhile, a similar cross-sectional study in Ghana by Obirikorang and Quaye [13] observed a sensitivity of 72.2%, specificity of 100.0%, PPV of 100.0%, and NPV of 95.7% at cutoff point < 1200cell/*μ*l in predicting CD4 count < 200 cell/*μ*l. TLC was observed as a better substitute for CD4 count in their study.

This study developed a combined marker comprising TLC, proteinuria, hematuria, and leukocyturia using the best cutoff points, and this yielded a better diagnostic accuracy (98.0%) and a good predictive performance for identifying HIV patients with CD4 count < 350 cell/*μ*l compared to the individual markers (Figure 1). This finding is novel and has not been reported elsewhere. The 98.0% diagnostic accuracy of the combined markers implied that only 2% will not need HAART. The significant associations of TLC, proteinuria, leukocyturia, and hematuria with CD4 count indicate that these markers play a synergistic role in the pathophysiology of HIV infections.

Although the findings of this study are novel, there were some limitations. We were not able to measure urine albumin-creatinine ratio to explain the significance of the proteinuria and how it relates to HIV-associated nephropathy (HIVAN). Despite our extensive effort to rule out confounding factors which may interfere with some dipstick findings like proteinuria, leukocyturia, and hematuria, there may be possible unforeseen confounders which were not controlled. However, we are confident in our findings and for the first time in Ghana, our study will be a baseline for further probing.

## 5. Conclusion

This study observed that proteinuria could serve as a noninvasive screening tool for predicting the severity of HIV infection. Meanwhile, a combination of total lymphocyte count, leukocyturia, proteinuria, and hematuria levels could serve as a substitute marker for CD4 count in resource-limited communities. It is therefore incumbent on the clinician to incorporate urine dipstick screening in routine HIV monitoring and management.

## Figures and Tables

**Table 1:** General characteristics of study participants.

Characteristics	Frequency	Percentages
*Age (year)*		
15–24	10	5.0
25–34	47	23.5
35–44	68	34.0
45–54	47	23.5
55–64	28	14.0
*Gender*		
Male	57	28.5
Female	143	71.5
*Marital status*		
Single	53	26.5
Married	35	17.5
Divorced	112	56.0

**Table 2:** Urine dipstick biochemistry and CD4, TLC, Hb, and WBC categorized by sex.

Characteristics	Total (*n* = 200)	Male (*n* = 57)	Female (*n* = 143)	*X* ^2^, df	*p* value
Mean age^b^	43.09 ± 10.33	43.44 ± 9.9	42.74 ± 10.76		0.5315
*Leucocytes* ^a^				3.580, 3	0.3105
Absent	100 (50.0%)	23 (40.3%)	77 (53.8%)		
1+	28 (28.0%)	9 (15.8%)	19 (13.3%)		
2++	48 (48.0%)	18 (31.6%)	30 (21.0%)		
3+++	24 (24.0%)	7 (12.3%)	17 (11.9%)		
*Leukocyturia*	100 (50.0%)	34 (34.0%)	66 (66.0%)		
*Protein* ^a^				4.503, 3	0.2121
Absent	97 (48.5%)	27 (47.4%)	70 (48.9%)		
1+	27 (13.5%)	12 (21.1%)	15 (10.5%)		
2++	51 (25.5%)	13 (22.8%)	38 (26.6%)		
3+++	25 (12.5%)	5 (8.8%)	20 (14.0%)		
*Proteinuria*	103 (51.5%)	30 (29.1%)	73 (70.9%)		
*Blood* ^a^				0.6404, 3	0.8871
Absent	91 (45.5%)	25 (43.9%)	66 (46.1%)		
1+	27 (13.5%)	9 (15.7%)	18 (12.6%)		
2++	57 (28.5%)	15 (26.3%)	42 (29.4%)		
3+++	25 (12.5%)	8 (14.0%)	17 (11.9%)		
*Hematuria*	109 (54.5%)	32 (29.4%)	77 (70.6%)		
Mean CD4 (cell/*μ*l)^b^	302.32 ± 32.4	273.43 ± 26.8	331.21 ± 38.0		0.1109
Mean TLC (cell/*μ*l)^b^	1166.59 ± 73.7	1121.83 ± 90.32	1211.36 ± 57.18		0.4017
Mean HB (g/dl)^b^	7.32 ± 1.64	7.12 ± 1.85	7.52 ± 1.43		0.1992
Mean WBC (×10^9^)^b^	2.36 ± 0.47	2.318 ± 0.51	2.403 ± 0.44		0.3629

Values are presented as mean ± SD and frequency (percentages). TLC: total lymphocyte count; Hb: hemoglobin; WBC: white blood cell. ^a^Chi-square test; ^b^Student's *t*-test.

**Table 3:** Association of CD4 count with urine dipstick biochemical analysis and TLC.

Characteristics	Total	CD4 count staging		CD4 < 350 cells/*μ*l	*p* value
		<350	≥350	aOR (95%C1)	
*Leukocyturia*					
Absent	100	8 (9.9%)	92 (77.3%)	Reference	
Present (≥+)	100	73 (90.1%)	27 (22.7%)	2.91(1.33 to 12.5)	<0.0001
*Proteinuria*					
Absent	91	1 (1.2%)	90 (75.6%)	Reference	
Present (≥+)	109	80 (98.8%)	29 (24.4%)	4.30 (3.0 to 18.5)	<0.0001
*Hematuria*					
Absent	97	10 (12.3%)	87 (73.1%)	Reference	
Present (≥+)	103	71 (87.6%)	32 (26.9%)	2.30 (1.08 to 9.64)	<0.0001
*TLC (cells/μl)*				Reference	
≤1200	107	69 (85.1%)	38 (31.9%)	3.26 (3.94 to 15.29)	<0.0001
>1200	93	12 (14.8%)	81 (68.1%)		

aOR: adjusted odds ratio for signs of active infections and conditions that may interfere with urine dipstick analysis.

**Table 4:** Individual diagnostic performance of selected urine dipstick biochemistry and TLC as alternative markers for CD4 Count.

Markers	Sensitivity	Specificity	PPV	NPV	AUC (*p* value)
*Leukocyturia*					0.901 (*p* < 0.0001)
≥1+	90.1%	77.3%	92.0%	73.1%	
**≥2++**	**92.1%**	**98.9%**	**100.0%**	**83.3%**	
≥3+++	68.3%	97.9%	88.70%	80.5%	
*Proteinuria*					0.973 (*p* < 0.0001)
≥1+	87.6%	73.1%	89.7%	68.9%	
**≥2++**	**97.6%**	**100.0%**	**100.0%**	**89.1%**	
≥3+++	73.0%	100.0%	99.8%	88.7%	
*Hematuria*					0.938 (*p* < 0.0001)
≥1+	98.7%	75.6%	98.9%	73.4%	
**≥2++**	**92.7%**	**98.6%**	**100.0%**	**83.7%**	
≥3+++	89.8%	93.1%	91.0%	85.2%	
*TLC (cell/μl)*					0.940 (*p* < 0.0001)
<800	76.3%	68.1%	82.9%	78.0%	
<1000	80.2%	74.8%	97.6%	84.3%	
**<1200**	**85.1%**	**100.0%**	**100.0%**	**88.9%**	
<1400	79.5%	87.2%	94.3%	72.3%	

**Table 5:** Combined diagnostic performance of urine dipstick biochemistry and TLC as alternative markers for CD4.

Markers	Sensitivity	Specificity	PPV	NPV	AUC (*p* value)
≤1200 TLC + leukocyturia ≥ 2++	76.2%	100.0%	100.0%	87.3%	0.937 (*p* < 0.0001)
≤1200 TLC + proteinuria ≥ 2++	88.1%	100.0%	100.0%	93.8%	0.978 (*p* < 0.0001)
≤1200 TLC + hematuria ≥ 2++	83.3%	100.0%	100.0%	89.2%	0.960 (*p* < 0.0001)
≤1200 TLC + ≥2++ (Leu + Pro + Hem)	72.8%	100.0%	100.0%	97.9%	0.980 (*p* < 0.0001)

Leu: leukocyturia; Pro: proteinuria; Hem: hematuria.

## References

[1] Piot P., Bartos M., Ghys P. D., Walker N., Schwartländer B. (2001). The global impact of HIV/AIDS. *Nature*.

[2] *Global AIDS Update 2017; July 2017. UNAIDS*.

[3] World Health Organization (2014). *Global Update on the Health Sector Response to HIV, 2014*.

[4] World Health Organization (2009). Towards universal access: scaling up priority HIV/AIDS interventions in the health sector.

[5] Sepkowitz K. A. (2001). AIDS — the first 20 years. *New England Journal of Medicine*.

[6] Kwamboka A. D., Juliette O., Amos M., Maturi P., Jackim N., Schifra U. (2016). Development of an algorithm of haematologic parameters as surrogate markers for CD4 cell count in resource-limited settings. *Journal of Medical Laboratory and Diagnosis*.

[7] Zachariah R., Ford N., Philips M. (2009). Task shifting in HIV/AIDS: opportunities, challenges and proposed actions for sub-Saharan Africa. *Transactions of the Royal Society of Tropical Medicine and Hygiene*.

[8] Awusabo-Asare K., Anarfi J. K. (1997). Health-seeking behaviour of persons with HIV/AIDS in Ghana. *Health Transition Review*.

[9] WHO Guidelines Approved by the Guidelines Review Committee (2016). *Consolidated guidelines on the use of antiretroviral drugs for treating and preventing HIV infection: recommendations for a public health approach*.

[10] van der Ryst E., Kotze M., Joubert G. (1998). Correlation among total lymphocyte count, absolute CD4+ count, and CD4+ percentage in a group of HIV-1-infected South African patients. *Journal of Acquired Immune Deficiency Syndromes and Human Retrovirology*.

[11] Alavi S. M., Ahmadi F., Farhadi M. (2009). Correlation between total lymphocyte count, hemoglobin, hematocrit and CD4 count in HIV/AIDS patients. *Acta Medica Iranica*.

[12] Daka D., Loha E. (2008). Relationship between total lymphocyte count (TLC) and CD4 count among peoples living with HIV, Southern Ethiopia: a retrospective evaluation. *AIDS Research and Therapy*.

[13] Obirikorang C., Quaye L., Acheampong I. (2012). Total lymphocyte count as a surrogate marker for CD4 count in resource-limited settings. *BMC Infectious Diseases*.

[14] Wondimeneh Y., Ferede G., Yismaw G., Muluye D. (2012). Total lymphocyte count as surrogate marker for CD4 cell count in HIV-infected individuals in Gondar University Hospital, Northwest Ethiopia. *AIDS Research and Therapy*.

[15] Akinola N. O., Olasode O., Adediran I. A. (2004). The search for a predictor of CD4 cell count continues: total lymphocyte count is not a substitute for CD4 cell count in the management of HIV-infected individuals in a resource-limited setting. *Clinical Infectious Diseases*.

[16] Sen S., Vyas A., Sanghi S. (2011). Correlation of CD_4+_ T cell count with total lymphocyte count, haemoglobin and erythrocyte sedimentation rate levels in human immunodeficiency virus type-1 disease. *Medical Journal Armed Forces India*.

[17] Morpeth S. C., Crump J. A., Shao H. J. (2007). Predicting CD4 lymphocyte count <200 cells/mm^3^ in an HIV type 1-infected African population. *AIDS Research and Human Retroviruses*.

[18] Kaswija J. P. (2010). *Proteinuria in ambulatory HIV-infected patients managed at Muhimbili national hospital (Doctoral dissertation, Muhimbili University of Health and Allied Sciences secondary school students)*.

[19] Amoako Y. A., Laryea D. O., Bedu-Addo G., Andoh H., Awuku Y. A. (2014). Clinical and demographic characteristics of chronic kidney disease patients in a tertiary facility in Ghana. *Pan African Medical Journal*.

[20] FolefackKaze F., Kengne A.-P., PefuraYone E. W., NdamFemben N. S., Ashuntantang G. (2013). Renal function, urinalysis abnormalities and correlates among HIV-infected Cameroonians naive to antiretroviral therapy. *Saudi Journal of Kidney Diseases and Transplantation*.

[21] Ryom L., Mocroft A., Kirk O. (2013). Association between antiretroviral exposure and renal impairment among HIV-positive persons with normal baseline renal function: the D:A:D Study^a^. *The Journal of Infectious Diseases*.

[22] Obirikorang C., Osakunor D. N. M., Ntaadu B., Adarkwa O. K. (2014). Renal function in Ghanaian HIV-infected patients on highly active antiretroviral therapy: a case-control study. *PLoS One*.

[23] Boucquemont J., Lawson-Ayayi S., Rigothier C. (2017). Absence of decline of kidney function in human immunodeficiency virus-infected patients under routine clinical management. *Nephron*.

[24] Fabian J., Naicker S., Venter W. D. (2009). Urinary screening abnormalities in antiretroviral-naive HIV-infected outpatients and implications for management—a single-center study in South Africa. *Ethnicity & Disease*.

[25] Cespedes R. D., Roettger R. H., Peretsman S. J. (1995). Herniation of the urinary bladder: a complication of traumatic pubic symphysis diastasis. *Southern Medical Journal*.

[26] Atta M. G., Choi M. J., Longenecker J. C. (2005). Nephrotic range proteinuria and CD_4_ count as noninvasive indicators of HIV-associated nephropathy. *The American Journal of Medicine*.

[27] Han T. M., Naicker S., Ramdial P. K., Assounga A. G. (2006). A cross-sectional study of HIV-seropositive patients with varying degrees of proteinuria in South Africa. *Kidney International*.

[28] Kamya M. R., Semitala F. C., Quinn T. C. (2004). Total lymphocyte count of 1200 is not a sensitive predictor of CD4 lymphocyte count among patients with HIV disease in Kampala, Uganda. *African Health Sciences*.

